# Pitch discrimination associated with phonological awareness: Evidence from congenital amusia

**DOI:** 10.1038/srep44285

**Published:** 2017-03-13

**Authors:** Yanan Sun, Xuejing Lu, Hao Tam Ho, William Forde Thompson

**Affiliations:** 1 Department of Cognitive Science, Macquarie University, Sydney, NSW, Australia; 2 ARC Centre of Excellence in Cognition and its Disorders, Sydney, NSW, Australia; 3 Department of Psychology, Macquarie University, Sydney, NSW, Australia; 4 CAS Key Laboratory of Mental Health, Institute of Psychology, Chinese Academy of Sciences, Beijing, China

## Abstract

Research suggests that musical skills are associated with phonological abilities. To further investigate this association, we examined whether phonological impairments are evident in individuals with poor music abilities. Twenty individuals with congenital amusia and 20 matched controls were assessed on a pure-tone pitch discrimination task, a rhythm discrimination task, and four phonological tests. Amusic participants showed deficits in discriminating pitch and discriminating rhythmic patterns that involve a regular beat. At a group level, these individuals performed similarly to controls on all phonological tests. However, eight amusics with severe pitch impairment, as identified by the pitch discrimination task, exhibited significantly worse performance than all other participants in phonological awareness. A hierarchical regression analysis indicated that pitch discrimination thresholds predicted phonological awareness beyond that predicted by phonological short-term memory and rhythm discrimination. In contrast, our rhythm discrimination task did not predict phonological awareness beyond that predicted by pitch discrimination thresholds. These findings suggest that accurate pitch discrimination is critical for phonological processing. We propose that deficits in early-stage pitch discrimination may be associated with impaired phonological awareness and we discuss the shared role of pitch discrimination for processing music and speech.

Music and language share many characteristics. Both involve a set of rules or principles through which constituents (tones in music and phonemes in language) are organized into complex, structured sequences^1^,^2^,^3^. Such similarities have led many to propose that music and language engage common cognitive resources^4^,^5^. This view is supported by evidence that skills specific to music correlate with phonological processing. In the case of individuals with normal language abilities, significant correlations have been reported between phonological awareness and melodic discrimination, pitch discrimination, pitch production, and meter perception^6^,^7^,^8^,^9^.

An association between musical abilities and phonological processing is also supported by reports of dyslexic individuals with concurrent phonological impairments^10^. Some studies suggest that these phonological impairments result from deficits at an early stage of auditory processing^11^,^12^,^13^, and may include deficits in frequency discrimination^14^ or temporal processing^15^,^16^. Phonological processing involves breaking down incoming speech stream into discrete sounds, mapping sounds to phonemes, and generating syllables and words. During this process, pitch changes as well as temporal variations (i.e., the amplitude envelop of the signal) within acoustic signals play important roles in establishing stress, segmenting speech stream, and interpreting sounds to meaningful speech units, such as phonemes, syllables and words^17^,^18^,^19^. Hence, effective auditory processing at an early, low-level stage of processing is essential for phonological processing. This early-stage auditory processing is not specialized for linguistic input, but acts upon all auditory input. As such, impairments at early-stages of auditory processing in dyslexics should have consequences for musical processing. Indeed, research has shown that children and adults with dyslexia display degraded performance in musical rhythm discrimination^20^,^21^, meter perception^22^, tapping to beats^20^,^23^, and/or pitch discrimination^24^,^25^. Furthermore, adults with dyslexia exhibit abnormal brain responses to changes in the pitch of tones, but not to changes in the duration of tones^26^.

In view of these findings, it is reasonable to hypothesize that individuals with poor musical abilities have parallel phonological deficits^7^. Indeed, it has been reported that individuals with deficits in music processing (“tune deafness”) have impaired phonological processing when compared with normal listeners^27^. However, tune deafness was determined by assessing the ability of participants to recognize wrong notes in popular melodies, and pitch and rhythm discrimination skills were not assessed. Therefore, it is unclear from this study whether the phonological impairments observed were related to deficits in pitch and rhythm processing.

Here, we asked whether deficits in pitch and rhythm processing are associated with language deficits, in particular, phonological processing. To answer this question, we tested participants who exhibit significantly impaired musical abilities (i.e., congenital amusia; which is comparable to tune deafness, but diagnosed differently), and their matched controls on several measures of phonological ability, including phonological awareness, phonological short-term memory and rapid naming^28^. Four subtests of the Comprehensive Test of Phonological Processing (CTOPP-2) were selected because they are considered to be valid and reliable measures of phonological processing ability^29^. A pure-tone pitch discrimination task and a rhythm discrimination task (see details in *Methods*) were administered in order to explore the relationship between these skills and phonological ability. If music processing and phonological processing draw upon the same auditory perceptual mechanisms^30^, then amusic participants should perform worse than control participants on the phonological tests.

## Results

### Pitch discrimination

In the pure-tone pitch discrimination task, we measured the just noticeable difference to discriminate pitches (i.e., pitch discrimination threshold). Participants were presented three tones, two of them were identical and the other one had a different pitch. Participants were then asked to identify the tone with the different pitch.

**Figure 1:**
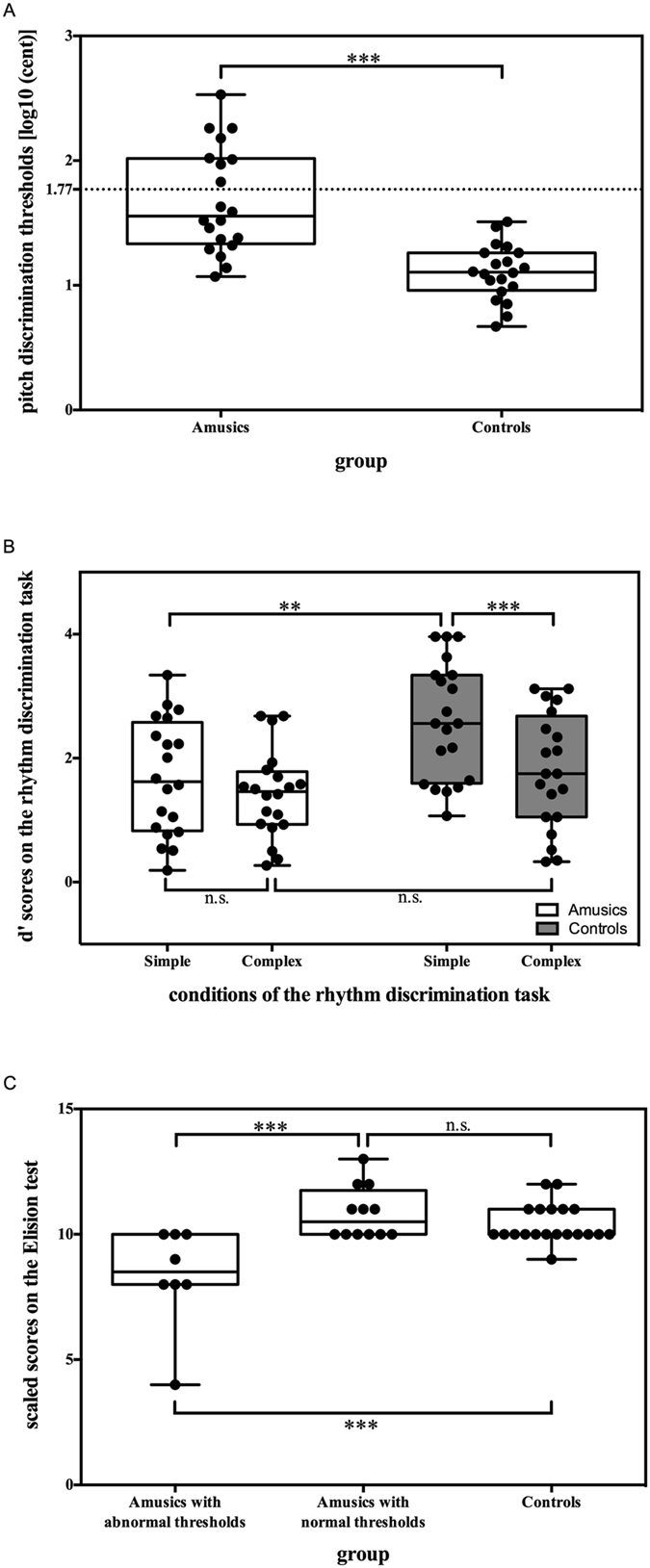
Participants’ performance on the (**A**) pitch discrimination task, (**B**) rhythm discrimination task, and (**C**) the Elision test. Boxplots show median values (middle horizontal line), 75^th^ percentile (box upper outline), 25^th^ percentile (box lower outline), maximum values (upper whiskers) and minimum values (lower whiskers). The dots overlaid with the boxplots represent the actual data points. The dotted line in Panel A shows three standard deviations (SD) above controls’ mean pitch discrimination thresholds. The asterisks indicate **p < 0.01; ***p <* 0.001*; and n.s. denotes ‘not significant’.

Raw pitch discrimination thresholds were calculated for each participant in cents (100 cents = 1 semitone). As the distribution of pitch discrimination thresholds is positively skewed especially for the amusia group (Skewness = 1.97; for the control group, Skewness = 1.12), raw threshold values were transformed to logarithm values with base 10 for further statistical analyses. The transformed pitch discrimination thresholds obtained for amusic and control participants were compared using an independent-samples t-test corrected for unequal variances. As can be seen in Fig. 1A, the mean pitch discrimination threshold was significantly higher for amusics [*M* = 76.27 cents, *SD* = 82.60 cents] than for controls [*M* = 14.38 cents, *SD* = 7.36 cents; *t* (38) = 5.37, *p* < 0.001, *Cohen*’*s d (hereafter d*) = 1.76]. Consistent with previous findings^31^,^32^, thresholds in the amusic group overlapped with those of the control group. However, eight of the 20 amusic participants showed exceptionally high pitch discrimination thresholds, that is, more than 3 SDs above the mean threshold observed for the control participants. The remaining amusic participants had pitch discrimination thresholds that were within the normal range.

### Rhythm discrimination

To assess how accurate the participants could discriminate rhythmic sequences, three rhythmic sequences were presented, each consisting of 5 to 7 pure tones. The first two sequences were identical in rhythm, and the third sequence was either identical to the first two or it differed rhythmically. Participants judged whether the third sequence was identical with the first two sequences. Two types of rhythmic sequences were created based on the complexity of the underlying metric structure – simple and complex (see details in *Methods*). Rhythmic sequences with a simple meter were constructed to induce clear and regular beats, whereas those with a complex meter were constructed to avoid inducing regular beats.

D-prime scores (henceforth d’) were calculated based on the hit and false alarm rate obtained on each condition (simple or complex meter) for each participant. A repeated-measures-ANOVA was conducted with the factors Rhythm (simple and complex meter) and Group (amusics and controls). Both Rhythm and Group showed a significant main effect [Rhythm: *F* (1, 38) = 29.09, *p *<* *0.001, 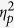 = 0.43; Group: *F* (1, 38) = 8.28, *p *=* *0.018, 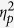
* *=* *0.14]. A significant interaction between Rhythm and Group was also found, [*F* (1, 38) = 7.37, *p* = 0.010, 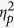
* *=* *0.16]. From Fig. 1B it can be seen that amusics performed worse than controls in the simple meter condition [amusics: *M* = 1.69, *SD* = 0.93; controls: *M* = 2.60, *SD* = 0.94; *F* (1, 38) = 9.50, *p *=* *0.004, 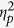
* *=* *0.20], however, this effect is mainly driven by six of the 20 amusics. Five of these six amusics also had abnormal pitch discrimination thresholds (see Supplementary Table 1). However, the group difference was not significant in the complex meter condition [amusics: *M* = 1.42, *SD* = 0.70; controls: *M* = 1.80, *SD* = 0.93; *F* (1, 38) = 2.10, *p *=* *0.156, 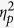
* *=* *0.05]. Looking only at the control group (Fig. 1B, grey boxes), we found that performance was significantly better when the meter was simple than when it was complex [*F* (1, 38) = 32.88, *p *<* *0.001, 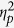
* *=* *0.46]. In contrast, for amusic participants there was only a marginally significant difference in performance between the simple and complex meter conditions [*F* (1, 38) = 3.59, *p *=* *0.066, 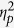
* *=* *0.09]. One-sample t-tests confirmed that both amusics and controls performed significantly above chance (a d’ score of zero) in both the simple [amusics: *t* (19) = 8.15, *p* < 0.001, *d* = 3.74; controls: *t* (19)* *=* *12.35, *p* < 0.001, *d* = 5.67] and complex [amusics: *t* (19) = 9.07, *p* < 0.001, *d* = 4.16; controls: *t* (19) = 8.67, *p* < 0.001, *d* = 3.98] meter conditions.

### Phonological tests

To evaluate the participants’ phonological abilities, we adopted four subtests from the CTOPP-2^29^. Phonological awareness was assessed by the Elision subtest, whereby participants were asked to repeat a word while omitting to vocalize certain syllables or phonemes. Phonological short-term memory was measured using the Non-Word Repetition and the Memory for Digits subtests, whereby participants repeated a number of nonsense words or random digit sequences. The rapid naming ability was evaluated using the Rapid Digit-Naming subtest, whereby participants read aloud a series of digits as fast as they can. Normalized composite scores derived from the CTOPP-2 were used in the statistical analyses.

We first compared the amusic and control participants’ performance on all administrated phonological tests. Independent-samples t-tests revealed no statistically significant group differences on any of the phonological tests (all *p*’s > 0.1). However, eight amusics with exceptionally high pitch discrimination thresholds (identified above) differed significantly from the other 12 amusics and the control participants in phonological processing. Given previous reports of a significant correlation between pitch and phonological processing (e.g., ref. 7), we compared performance on phonological awareness for the three groups (amusics with abnormal pitch discrimination thresholds, amusics with normal pitch discrimination thresholds, and control participants). The analysis revealed a significant group difference on the Elision test, which measures phonological awareness (see Table 1). Post hoc tests with Bonferroni correction revealed that amusics with abnormally high pitch discrimination thresholds performed significantly worse than amusics with normal pitch discrimination thresholds (*p* < 0.005) and worse than the controls (*p *=* *0.001), as shown in Fig. 1C. There was no significant difference observed between amusics with normal pitch discrimination thresholds and control participants (*p *=* *0.947). Finally, no group differences were observed for other phonological tests (all *p*’s > 0.05), as shown in Table 1.

To further examine the 8 amusics with abnormal pitch discrimination thresholds, we next considered melodic MBEA scores, IQ scores, and reading scores (see details in *Methods*) for the 8 amusics with abnormal pitch discrimination thresholds, the 12 amusics with normal pitch discrimination thresholds, and control participants. Amusics with abnormal pitch discrimination thresholds performed similarly to control participants on the IQ and reading tests, but worse than the controls on the melodic MBEA (refer to Table 2 for details). No group differences between amusics with and without abnormal pitch discrimination thresholds were observed in these three tests (all *p*’s >0.05).

### Regressions

**Figure 2:**
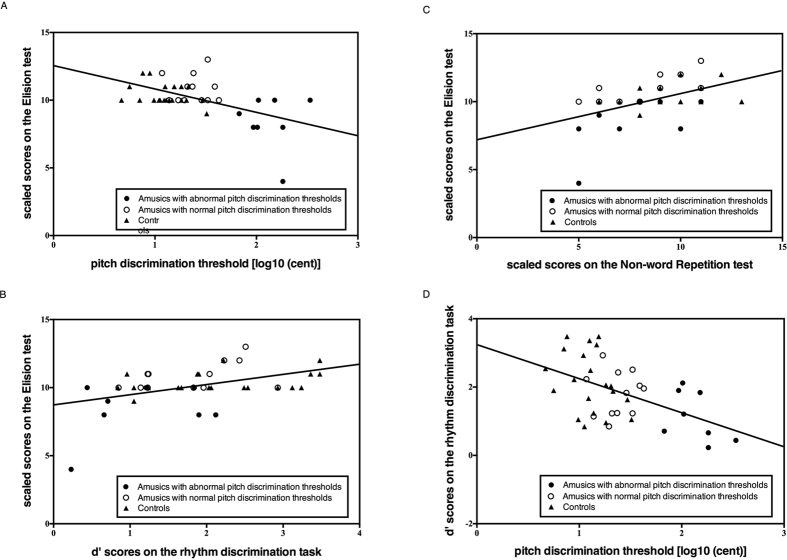
Results of correlational analyses across all participants. Elision test performance significantly correlated with (**A**) the pitch discrimination thresholds; (**B**) composite d’ scores on the rhythm task; and (**C**) scores on the Non-Word Repetition test. After removing the outlier (having Elision score of 4), the Elision test still significantly correlated with pitch discrimination thresholds [*r* (37) = −0.453, *p* = 0.004], rhythm discrimination [*r* (37) = 0.362, *p* = 0.024] and Non-Word Repetition test [*r* (37) = 0.446, *p* = 0.004]. (**D**) Pitch discrimination thresholds strongly correlated with scores on the rhythm task. Black and white circles represent amusic participants with abnormal and normal pitch discrimination thresholds respectively. Controls are represented by black triangles. The depicted lines are based on linear regressions.

The results of three Pearson correlation analyses (2-tailed) indicated that phonological awareness was significantly correlated with (i) pitch discrimination ability [*r* (38) = −0.53, *p* < 0.001], (ii) rhythm discrimination ability [simple condition, *r* (38) = 0.42, *p* = 0.007; complex condition *r* (38) = 0.44, *p* = 0.004; combining simple and complex conditions, *r* (38) = 0.46, *p* = 0.003] and (iii) phonological short-term memory as measured by the Non-Word Repetition test [*r* (38) = 0.50, *p* = 0.001]. Scatter plots of these relationships are displayed in Fig. 2A–C. Correlations between phonological awareness and other administrated tests (i.e., non-verbal IQ, Reading, Memory for Digits, Rapid Digit-Naming; see *Methods* for details) were not significant (all *p*’*s* >0.05). In order to confirm whether individual differences in pitch discrimination ability make a unique contribution to the prediction of phonological awareness, we employed a hierarchical regression analysis. Given that measures of both simple and complex rhythm discrimination were correlated with phonological awareness, a composite score that considered these two rhythmic conditions was created and used in the regression analysis. The regression analysis also included a predictor based on the interaction between pitch and rhythm measures, reflecting the significant association between pitch and rhythm discrimination observed in this study [*r* (38) = −0.51, *p* = 0.001; refer to Fig. 2D and also see above subsection].

Prior to conducting the multiple regression, the relevant regression assumptions for regression analysis were tested, including the assumptions of no outliers, collinearity, independent errors, normally distributed errors, homogeneity of variance and linearity, and non-zero variances^33^. The results confirmed that the data met all above assumptions. Subsequently, a hierarchical multiple regression was performed across all participants (N = 40) using a four-step fixed entry equation with phonological awareness (the scores on the Elision test) as the dependent variable. The predictors were: Step 1) measures on the Non-Word Repetition test; Step 2) rhythm discrimination measures; Step 3) interaction between pitch and rhythm discrimination; Step 4) measures of pitch discrimination thresholds. As shown in Table 2, the measure of the Non-Word Repetition task can significantly predict phonological awareness (*β *=* *0.50, *p *=* *0.001) with 25% explained unique variance. Adding the measure of rhythm discrimination into the regression model explained an additional 9% of unique variance and this change in *R*^*2*^ was significant (*β *=* *0.33, *p *=* *0.028). However, the model did not perform significantly better when the interaction between pitch and rhythm discrimination was included (*β *=* *−0.25, *p *=* *0.244). Finally, the addition of pitch discrimination to the regression model explained an additional 10% of the variance in phonological awareness (*β *=* *−0.65, *p *=* *0.013). However, rhythm discrimination was no longer significant (*β *=* *−0.36, *p *=* *0.365) when pitch discrimination was entered into the regression model.

## Discussion

This investigation examined pitch discrimination, rhythm discrimination, and phonological abilities, as well as the associations between these measures, in individuals with and without congenital amusia. Among other characteristics, amusia is associated with an impaired ability to perceive small pitch differences^34^,^35^. At the group level, the amusic participants in our study had significantly higher pitch discrimination thresholds than those of controls. Close inspection of the data, however, revealed a substantial overlap in the distribution of pitch discrimination thresholds across groups, consistent with previous investigations^31^,^32^. In other words, not all amusics, as identified by the melodic Montreal Battery of Evaluation of Amusia^36^ (MBEA) tests, exhibited high pitch discrimination thresholds. Instead, some amusics performed in the normal range exhibited by control participants.

Eight of the 20 amusic participants had severe difficulty discriminating an odd tone from two standard tones, especially when the pitch difference was less than 50 cents. We observed that these eight amusics, who had exceptionally high pitch discrimination thresholds, also performed poorly on the Elision test. The Elision test measures phonological awareness by requiring participants to engage in speech-sound manipulations (e.g., say “cup” without the phoneme/k/). In contrast, the other 12 amusics with relatively normal pitch discrimination thresholds performed as well as controls on the Elision test. The results of the regression analysis suggest that pitch discrimination thresholds can predict phonological awareness, even when we take into account the variances shared with phonological short-term memory and rhythm discrimination. This finding implies that pitch discrimination ability has a reliable and unique relationship with phonological awareness.

The abnormal pitch discrimination thresholds exhibited by the eight amusic participants identified in this study may reflect a problem in early-stage pitch processing, as suggested by Peretz *et al*.^34^. Pitch discrimination relies on acoustic pitch encoding, which occurs in the ascending auditory pathway and up to the primary auditory cortex^37^. Recent studies have traced the source of pitch deficits in congenital amusia to early brain responses in the auditory cortex^38^, or in the brainstem^39^ (but see ref. 40). These early-stage impairments may then have a cascade effect upon later stages^34^, regardless of the domain (music or language). Indeed, other research on congenital amusia suggests that an early-stage pitch deficit can significantly affect late-stage pitch-related language processing, such as speech intonation^32^, lexical tone^41^ and emotional speech prosody^42^. Although the phonological awareness task does not have an obvious link with pitch discrimination, pitch-based prosodic cues assist in the process of segmenting a continuous speech stream into phonemes and syllables^43^. Therefore, when early-stage pitch processing is impaired, phonological discrimination, segmentation and blending (the abilities tested in the Elision test) may be affected. The remaining 12 amusics appear to have intact early-stage pitch processing, given their normal pitch discrimination thresholds. These individuals still performed poorly on the melodic MBEA tests, however, suggesting that their musical deficits are associated with a late stage of processing, such as memory for musical pitch^44^,^45^,^46^. These late-stage impairments may be music-specific and independent of late-stage language processing.

Other early stage deficits in auditory processing should also affect both musical and phonological processing. For example, deficits in echoic memory should lead to poor performances in both the pitch discrimination task and the Elision test. However, we observed no differences in phonological short-term memory either between amusics and control participants, or between amusics with and without abnormal pitch discrimination thresholds. Thus, the only early stage impairment observed among selected amusics was pitch discrimination, and this impairment appears to be the source of correlated performance between music and phonological processing.

In our study, all participants with high pitch discrimination thresholds were also classified as amusics. In a more general population, however, some individuals with high pitch discrimination thresholds may not be identified as amusics according to the diagnostic criteria outlined for the MBEA. Therefore, it is not possible to conclude from the current results that a high pitch discrimination threshold was solely responsible for reduced performance on the Elision test. The phonological impairment observed in the Elision test could relate to pitch deficits, or it could be the result of an interaction between pitch deficits and other characteristics of congenital amusia. Future studies are needed to confirm whether individuals with high pitch discrimination thresholds but normal music perception skills (as indicated by, for example, the MBEA^36^) exhibit impairments in phonological processing.

In addition to pitch impairments, we found that amusics performed worse than controls in rhythm discrimination, but only when the metric structure was simple. However, it is worth noting that the group difference on the simple meter condition was primarily driven by the performance of six amusics, who all performed extremely poorly. Our observation that poor rhythm perception is only observed in some amusics is consistent with previous findings^35^. According to Foxton and colleagues^47^, rhythm deficits exhibited by amusics often depend on the presence of pitch variation in the melodies, as exemplified by the MBEA rhythm subtest. Thus, amusics may perform normally on rhythm tests involving fixed-pitch (monotonic) sequences (see also ref. 48), but abnormally on rhythm tests involving pitch-varying sequences. Nonetheless, using monotonic rhythmic sequences, 30% of amusic participants in our study showed clear impairment in discriminating simple rhythmic patterns, which implies that a subgroup of amusic individuals have a rhythm impairment that cannot be attributed to pitch changes within sequences.

These findings contrast with two studies showing that amusics perform as well as controls on monotonic rhythmic sequences^47^,^48^. One possible reason for this discrepancy may relate to differences in the rhythmic sequences employed. Specifically, the sequences employed by Hyde and Peretz^48^ and Foxton and colleagues^47^ consisted of tones of equal duration, with changes restricted to the *inter-tone interval* (period of silence between tones). In our sequences, the duration of the tones varied, while the inter-tone intervals were held constant (40 ms). Grahn and Brett^49^ introduced this strategy of assessing rhythm processing for the purpose of assessing auditory capabilities in individuals with Parkinson’s disease. Arguably, the approach may also be appropriate for identifying deficits in temporal processing in individuals with congenital amusia.

For complex metric structures, amusics and controls showed no significant group difference: both groups exhibited difficulty in the discrimination of complex rhythmic structures. Simple rhythms induced the perception of regular beats, whereas no such regularity could be extracted from complex rhythmic structures. The control group performed significantly better in the simple rhythm condition than in the complex rhythm condition, suggesting that they benefited from the regularity underlying a simple metric structure. However, the amusia group benefitted only marginally from the regularity underlying a simple metric structure, leading them to perform more poorly than the control group on this condition. As shown in Fig. 1B, six amusics had difficulty extracting the regular cycle of stress in music that typical listeners perceive.

Given the rhythm impairment observed among our amusic participants, we asked whether participants’ rhythm ability might be correlated with their phonological ability. A number of studies suggest that rhythm discrimination is associated with phonological processing^22^,^23^,^50^,^51^,^52^,^53^. Corroborating these results, we observed a significant correlation between rhythm discrimination and phonological awareness. However, a further regression analysis revealed that rhythm discrimination sensitivity does not remain a significant predictor in a regression model once pitch discrimination was taken into account. This implies that the association between rhythm discrimination and phonological awareness observed in the present study was driven by the association between rhythm discrimination and pitch discrimination. Indeed, the amusic participants with abnormally high pitch discrimination thresholds also had difficulty discriminating simple rhythmic sequences.

Pitch and rhythm are often considered as independent dimensions of music^54^. Support for independence between pitch and rhythm comes from findings that certain individuals exhibit rhythm-related musical impairments but have intact pitch perception, including individuals who suffer beat deafness^55^ and dysrhythmia^56^. Thus, it is possible that the pitch and rhythm difficulties exhibited by the 8 amusics in our investigation may be unrelated to each other. Alternatively, these amusics may have a general auditory deficit that affects both pitch and rhythm processing. For example, research suggests that impaired encoding of rapid auditory information is sometimes associated with impaired pitch discrimination in congenital amusia^57^. Thus, it is possible that deficits in rapid auditory processing might lead to poor performance in both pitch and rhythm tasks.

## Conclusion

We investigated the association between music perception (pitch and rhythm discrimination) and phonological abilities (phonological awareness, phonological short-term memory, and rapid-naming) in individuals with and without congenital amusia. Our results suggest that congenital amusia is not generally associated with phonological deficits. However, a subgroup of amusics with very high pitch discrimination thresholds did exhibit impairments in phonological awareness. This association was confirmed by regression analyses, which showed that pitch discrimination thresholds could predict phonological awareness even when sensitivity to rhythm was statistically controlled. In contrast, sensitivity to rhythm was not a significant predictor of phonological awareness once pitch discrimination thresholds were statistically controlled. As suggested by previous studies^34^,^37^,^38^, pitch discrimination may reflect an early-stage auditory function. The observed association between high pitch thresholds and phonological awareness implies that an impairment at an early stage of pitch encoding can negatively impact on both music and language processing. It should be noted that deficits in other cognitive processes such as working memory, attention, or decision-making may also lead to poor performance in a pitch discrimination task. As a future direction, this possibility could be explored using electrophysiological measures such as EEG. With its high temporal resolution, EEG would allow investigators to determine whether amusics exhibit abnormal brain activity at early stages of pitch processing and phonological processing. More generally, determining the sources of phonological impairments in amusia would contribute to a better understanding of language disorders such as dyslexia.

## Methods

### Participants

To identify individuals with congenital amusia, three melodic subtests (Scale, Contour and Interval) of the MBEA^36^ were administered as screening tests, which are considered more effective in identifying congenital amusics (henceforth amusics) than the rhythm and meter subtests (i.e., only 50% of individuals with amusia have impaired rhythm processing^36^). In each subtest, listeners were presented with pairs of melodies and asked to judge whether the melodies were the same or different. Participants who scored at or below 72.22% across the three subtests (i.e., 65 out of 90 points) were identified as amusics. D-prime has been suggested to use in identifying amusics^58^,^59^, which is more conservative. However, this study aimed to assess the general association between musical skills and phonological abilities, and not in the diagnosis of congenital amusia *per se*. Therefore, we applied the accuracy here as previous studies used^32^,^42^,^60^. In order to exclude dyslexic participants, any participant who scored more than 1.5 SD below the norm mean of the composite index on the second edition of the Test of Word Reading Efficiency (TOWRE-2)^61^ was not included. To ensure participants were comparable in general cognitive processing, non-verbal intelligence was also assessed using the Matrices subtest of the Kaufman Brief Intelligence Test II (KBIT-2)^62^.

Twenty monolingual Australian English speakers aged 18–24 with poor musical abilities (13 females) and 20 matched controls (13 females) completed these tests. All participants were right-handed^63^, and had normal hearing (<30 dB) in both ears at the frequencies 0.25, 0.5, 1, 2, 4, and 8 kHz, as measured by the Otovation Amplitude T3 series audiometer (Otovation LLC, PA, United States). None of the individuals reported having a past history of neurological or psychiatric disorders. There were no significant differences between two groups in age, years of education, years of musical training, reading ability, or non-verbal IQ (all *p*’*s* > 0.05, see details in Table 3). All participants provided written informed consent and received payment or course credit points for their time. This study was carried out in accordance with approved guidelines of the National Statement on Ethical Conduct in Human Research (2007). Ethical approval was obtained from the Ethics Committee of Macquarie University.

### Tasks

#### Pitch discrimination

In the pitch discrimination threshold task, participants heard three pure tones centred at 500 Hz for each trial. Each tone was 500 ms in length and had a 10 ms onset and offset ramp. The three tones were presented in random order with an inter-stimulus interval of 500 ms. Two of the three tones had an identical pitch (i.e., standard tones), whereas the other tone (i.e., deviant tone) was either higher or lower in pitch than the standard tones. The task used an adaptive “3-down 1-up” staircase paradigm^64^ implemented in the STAIRCASE toolbox^65^. Participants were required to indicate via a key press which of the three tones was different from the other two tones, using a 3-alternative forced choice task. After three consecutive correct responses there was a decrease in pitch excursion, whereas every incorrect response resulted in an increase in pitch excursion. The initial (default) pitch difference was 6 semitones. For adaptive pitch changes, the initial change in step size was 1 semitone, but this change was reduced to 0.1 semitones after four reversals, and then reduced to 0.02 semitones after eight reversals. Participants received feedback after each trial. The task was terminated after 14 track reversals and the threshold was calculated as the arithmetic mean differential value of the pitch intervals for the last 6 reversals.

#### Rhythm discrimination

**Figure 3:**
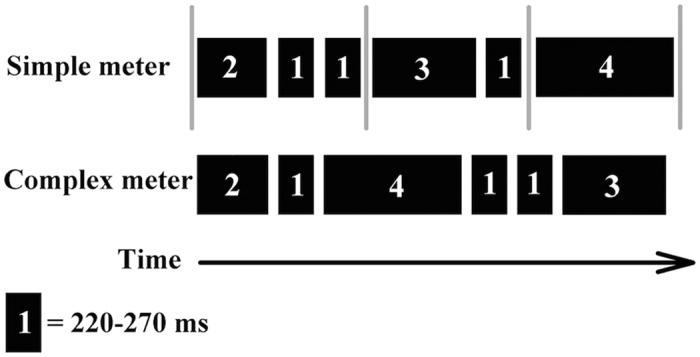
An illustration of the rhythm discrimination task based on the diagram of Grahn and Brett^49^. The black boxes indicate the duration of each tone. The number in each black box represents the relative duration of the tone in each sequence, with 1 = 220–270 ms. The tone duration was randomly chosen on each trial in steps of 10 ms, and followed a ratio of 1:2:3:4. Grey bars indicate the regular metric structure, which can only be perceived in the simple meter condition.

To assess rhythm ability in the absence of pitch changes, we presented participants with rhythmic sequences consisting of pure tones at a constant frequency throughout a trial. Rhythmic sequences were constructed in accordance with those described by Grahn and Brett^49^. The fixed pitch was randomly selected from a pre-defined pitch range of 294 Hz (D4) to 587 Hz (D5). The rhythmic sequences comprised 5 to 7 tones, with adjacent tones separated by a 40 ms period of silence. As illustrated in Fig. 3, the durations of the tones were related to each other by ratios of 1:2:3:4. The duration of the shortest tone (marked ‘1’ in Fig. 3) was randomly selected from six possible durations (220, 230, 240, 250, 260, and 270 ms) with an 8 ms ramp. The duration of the other tones were integer multiples of the shortest tone. The rhythmic sequences were grouped into two types of meters – simple and complex (see Fig. 3). The tones in the simple meter condition were arranged in such a way that the emerging rhythm was perceived as having a quadruple meter. The tones in the complex meter condition were the same as those in the simple meter condition; however, their positions were scrambled so that the emerging rhythm did not give rise to the perception of a regular meter as in the simple meter condition.

Each trial comprised three rhythmic sequences presented with a silent inter-sequence interval of 1100 ms. The first two sequences were identical (i.e., *the standard*), whereas the third sequence (i.e., *the comparison*) could have the same or a different rhythm. Participants were asked to indicate via key press whether or not the third rhythm was identical to the previous two sequences. Unlike the block design used by Grahn and Brett^49^, simple and complex rhythm conditions were presented in the same block and the order of trials was scrambled. Before commencing the experiment, each participant completed a practice block of 8 trials, which were randomly selected from the set of 120 experimental trials. In half of the trials, the standard and comparison rhythms were identical. In the other half, the standard and comparison rhythms differed by transposing sequences, but maintaining the same metric structure, that is, simple or complex. For instance, the sequence of durations 211314 (as shown in Fig. 3) might be re-ordered to yield a different sequence of durations 211134, such that both the standard and comparison sequences were consistent with a simple meter. The auditory stimuli were delivered using a NVIDIA soundcard and controlled using the software Presentation, Version 16.4 (Neurobehavioral Systems Inc., CA, United States).

#### Phonological tests

We applied four subtests of CTOPP-2^29^ to test the participants’ phonological abilities. The Elision test was used to assess phonological awareness, which is designed to assess an individual’s ability to mentally segment compound and non-compound words into their phonological components (e.g., toothbrush, winter), imagine the words with specific elements omitted, and re-assemble the segments into new words. We first recorded the original items of the Elision test with a female native Australian English speaker on CD. The resultant stimuli were delivered via built-in loudspeakers on a MacBook Pro (Apple Inc., CA, United States). Participants were asked to repeat each word but with one syllable or one phoneme removed, e.g., ‘toothbrush’ without ‘tooth’ and ‘winter’ without the phoneme/t/. The test comprises 34 items, nine of which are compound words, while the rest are non-compound words. The test was terminated when participants failed to perform the Elision test on three trials in a row. Successful trials were then counted and the sum converted to a normalised score for assessment.

The Memory for Digits test and Non-Word Repetition test were conducted to assess subjects’ phonological short-term memory. In order to assess participants’ memory for digits, 28 number sequences were presented. For this test, we used the CD recording provided with the CTOPP-2 battery. Participants were asked to repeat the digits in the same order as they heard them. The length of the sequences increased from two to nine digits as participants repeated a sequence correctly. The test ended when participants made three errors in a row. Again, the number of successful trials was converted to a normalised score for assessment. Non-Word Repetition was assessed using the audio recordings provided with the CTOPP-2 battery. Participants listened to 30 non-words (e.g. ‘wudoip’), which they had to repeat. The length of the non-words increased from 3 to 15 phonemes as participants repeated each item correctly. The test ended when participants failed to correctly repeat three items in a row. A normalised score was obtained from converting the number of successful trials according to a scale provided by the CTOPP-2 battery.

Finally, rapid naming was assessed using the Rapid Digit-Naming test. In this task, participants were presented with 36 digits printed on a paper card and asked to name the digits as fast as possible. This test requires rapid retrieval of phonological representations of digits from long-term memory. The time it takes participants to name all 36 digits was recorded and converted to a normalised scale provided by the CTOPP-2 battery for assessment.

#### Procedures

Participants were tested individually in a quiet testing room on all the administrated tasks. The tasks were conducted in a fixed order: (i) pitch discrimination, (ii) rhythm discrimination, and (iii) phonological tests. The phonological tests were administrated according to standardised instructions that accompanied each subtest. Participants’ voice responses were digitally recorded, and subsequently evaluated.

## Additional Information

**How to cite this article**: Sun, Y. *et al*. Pitch discrimination associated with phonological awareness: Evidence from congenital amusia. *Sci. Rep.* **7**, 44285; doi: 10.1038/srep44285 (2017).

**Publisher's note:** Springer Nature remains neutral with regard to jurisdictional claims in published maps and institutional affiliations.

## Supplementary Material

Supplementary Table 1

## Figures and Tables

**Table 1:** Performance of the two amusic subgroups and the control group on the administrated four phonological measures.

Phonological measures	Amusics with abnormal pitch discrimination thresholds (n = 8)	Amusics with normal pitch discrimination thresholds (n = 12)	Controls (n = 20)	Statistics		
	*M (SD*)	*M (SD*)	*M (SD*)	*F*	*p*	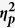
Elision	8.38 (2.00)	10.83 (1.03)	10.40 (0.75)	1.77	<0.001	0.39
Memory for Digits	10.13 (3.72)	9.58 (2.97)	11.00 (1.86)	0.13	0.335	0.06
Non-Word Repetition	7.63 (2.26)	8.17 (1.94)	9.15 (2.11)	0.80	0.179	0.09
Rapid Digit-Naming	11.25 (2.66)	10.92 (1.68)	10.50 (1.47)	0.54	0.586	0.03

The *F*-values, *p*-values and 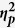 were obtained from ANOVAs. *M* = mean and *SD* = standard deviation.

**Table 2:** Characteristics and pre-test scores of control participants, amusic participants, and the amusic participants divided into two subgroups.

	Controls (n = 20)	Amusics (n = 20)	Amusics with abnormal pitch discrimination thresholds (n = 8)	Amusics with normal pitch discrimination thresholds (n = 12)
	*M (SD*)	*M (SD*)	*M (SD*)	*M (SD*)
Age (years)	19.94 (1.84)	20.56 (2.29)	20.55 (2.47)	20.58 (2.27)
Education (years)	14.23 (1.46)	14.58 (2.40)	15.19 (3.09)	14.17 (1.85)
Musical training (years)	0.75 (1.21)	0.30 (0.92)	0.13 (0.35)	0.42 (1.16)
IQ (scaled score)	105.95 (11.66)	100.45 (10.36)	101.25 (13.51)	99.92 (8.27)
Reading (scaled score)	104.05 (7.21)	103.20 (9.60)	103.38 (11.90)	103.08 (8.31)
Melodic MBEA (%)				
Scale	91.33 (5.56)	73.67 (12.14)	73.75 (13.02)	73.61 (12.10)
Contour	83.83 (7.11)	62.83 (7.82)	60.42 (6.77)	64.45 (8.33)
Interval	83.00 (8.01)	63.16 (8.62)	64.58 (6.89)	62.22 (9.78)
Composite	86.89 (5.03)	66.55 (4.47)	66.25 (4.89)	66.76 (4.38)

**Table 3:** Hierarchical Regression of the phonological awareness measures in four-step fixed entry regression equations (n = 40).

Predictors	Step 1			Step 2			Step 3			Step 4		
	*B*	*SE*	*β*	*B*	*SE*	*β*	*B*	*SE*	*β*	*B*	*SE*	*β*
Non-Word	0.34	0.10	0.50**	0.26	0.10	0.39*	0.27	0.10	0.39**	0.22	0.09	0.33*
Rhythm				0.54	0.24	0.33*	0.87	0.36	0.52*	−0.60	0.65	−0.36
Pitch × Rhythm							−0.36	0.30	−0.25	0.67	0.48	0.48
Pitch										−2.13	0.81	−0.65*
*R*^*2*^	0.23			0.31			0.31			0.41		
*Δ R*^*2*^	0.25**			0.09*			0.03			0.10**		
*F*	12.51**			9.55***			6.90**			7.76***		

Non-Word = Non-Word repetition test scores; rhythm discrimination = composite d’ scores combining the two rhythm conditions (simple and complex rhythmic patterns); Pitch = pitch discrimination thresholds; Pitch × Rhythm = the interaction between pitch and rhythm discrimination. *B* = unstandardised B; *SE* = coefficients standard errors; *β *=* *standardised beta coefficient; *R*^*2*^ = adjusted R square*; Δ R*^*2*^* *=* R*^*2*^ change; *p < 0.05, **p <* *0.01, ***p < 0.001.
