# Supplementary material for: Pitch discrimination associated with phonological awareness: Evidence from congenital amusia
Source: Sci Rep. 2017 Mar 13;7:44285. doi: 10.1038/srep44285 (PMC5347159; doi:10.1038/srep44285)
Supplement: Supplementary Table 1 [file srep44285-s1.pdf]

Pitch discrimination associated with phonological awareness: Evidence from congenital amusia

Yanan Sun[1,2,*], Xuejing Lu[2,3,4], Hao Tam Ho[2,3], William Forde Thompson[2,3]

[1] Department of Cognitive Science, Macquarie University, Sydney, NSW, Australia

[2] ARC Centre of Excellence in Cognition and its Disorders, Sydney, NSW, Australia

[3] Department of Psychology, Macquarie University, Sydney, NSW, Australia

[4] CAS Key Laboratory of Mental Health, Institute of Psychology, Chinese Academy of Sciences, Beijing, China

* **Correspondence:** Yanan Sun, Department of Cognitive Science, Australian Hearing Hub, 16 University Avenue, Macquarie University, NSW, 2109, Australia. Email: yanan.sun@mq.edu.au

Supplementary information

Group[1]	ID	Melodic MBEA[2]				IQ[3]	Reading[4]	Pitch	Rhythm[6]			Phonology[7]			
		Scale	Contour	Interval	Composite			Thresholds[5]	Simple	Complex	Composite	Elision	Digits	Non-word	RAN
A1	A101	60.00	66.67	73.33	66.67	100	91	2.26	0.81*	0.50	0.66	8	9	7	8
A1	A102	70.00	70.00	66.67	68.89	103	102	1.83	0.54*	0.88	0.71	9	10	6	12
A1	A103	80.00	56.67	66.67	67.78	86	98	2.26	0.19*	0.27	0.23	4	5	5	13
A1	A104	50.00	60.00	60.00	56.67	95	120	2.18	2.22	1.54	1.84	10	9	11	14
A1	A105	80.00	50.00	73.33	67.78	132	113	2.02	0.88*	1.58	1.21	10	15	9	12
A1	A106	86.67	63.33	60.00	70.00	100	102	1.97	2.68	1.40	1.90	8	10	5	10
A1	A107	76.67	53.33	53.33	61.11	94	86	2.53	0.51*	0.37	0.44	10	7	8	7
A1	A108	86.67	63.33	63.33	71.11	100	115	2.01	2.86	1.53	2.12	8	16	10	14
A2	A201	73.33	76.67	60.00	70.00	96	104	1.63	2.01	1.93	1.96	10	7	8	9
A2	A202	86.67	56.67	73.33	72.22	115	113	1.07	2.78	1.81	2.23	12	13	10	12
A2	A203	50.00	56.67	83.33	63.33	96	112	1.52	2.36	2.68	2.51	13	13	11	12
A2	A204	63.33	63.33	53.33	60.00	105	95	1.46	1.67	1.70	1.83	10	6	7	12
A2	A205	60.00	80.00	53.33	64.44	107	111	1.14	1.14	1.14	1.14	10	12	6	12
A2	A206	73.33	66.67	53.33	64.44	98	92	1.37	1.50	1.09	1.24	11	9	6	7
A2	A207	76.67	70.00	63.33	70.00	108	102	1.23	3.34	2.61	2.93	10	12	8	12
A2	A208	70.00	53.33	56.67	60.00	98	114	1.32	1.05	1.50	1.23	11	11	9	13
A2	A209	73.33	70.00	53.33	65.56	100	96	1.38	2.23	2.68	2.43	12	6	9	10

A2	A210	86.67	60.00	60.00	68.89	82	101	1.59	2.65	1.42	2.04	11	12	11	10
A2	A211	93.33	60.00	63.33	72.22	99	106	1.29	0.77*	0.94	0.85	10	9	8	11
A2	A212	76.67	60.00	73.33	70.00	95	91	1.52	1.57	0.93	1.23	10	5	5	11
C	C101	93.33	86.67	90.00	90.00	111	98	0.85	3.12	3.12	3.12	10	12	8	9
C	C102	86.67	80.00	90.00	85.56	103	112	0.75	2.46	1.42	1.90	11	11	11	9
C	C103	80.00	73.33	73.33	75.56	103	110	1.47	2.75	0.77	1.63	10	10	7	11
C	C104	93.33	90.00	73.33	85.56	130	102	0.95	3.34	1.50	2.22	12	11	12	10
C	C105	100.00	90.00	76.67	88.89	90	92	1.05	1.58	0.35	0.84	10	8	11	10
C	C106	86.67	90.00	73.33	83.33	97	103	1.17	3.24	2.94	3.24	10	10	6	10
C	C107	96.67	70.00	76.67	81.11	95	101	0.99	1.49	0.33	1.05	10	11	6	9
C	C108	100.00	76.67	83.33	86.67	120	108	1.31	2.12	2.09	2.03	10	11	11	13
C	C109	90.00	90.00	90.00	90.00	100	99	1.19	3.96	3.12	3.48	11	11	11	9
C	C110	90.00	76.67	76.67	81.11	98	104	1.26	1.46	0.52	0.96	11	9	9	10
C	C111	96.67	90.00	90.00	92.22	111	116	1.11	2.56	2.47	2.49	10	15	10	11
C	C112	83.33	90.00	83.33	85.56	104	111	1.04	3.96	2.34	2.93	10	11	9	12
C	C113	93.33	80.00	86.67	86.67	103	106	1.26	2.56	1.75	2.06	10	9	9	12
C	C114	86.67	83.33	80.00	83.33	95	102	1.10	3.63	2.75	3.36	11	12	11	13
C	C115	93.33	83.33	80.00	85.56	100	115	1.51	1.07	1.05	1.05	9	11	8	13
C	C116	96.67	93.33	100.00	96.67	130	90	0.88	3.96	3.00	3.48	12	16	10	8
C	C117	93.33	76.67	80.00	93.33	98	111	1.14	1.53	1.05	1.24	10	11	13	10
C	C118	90.00	86.67	73.33	90.00	120	95	1.09	1.64	1.75	1.67	10	11	6	11

C	C119	83.33	93.33	96.67	83.33	96	102	0.67	3.34	2.12	2.54	10	9	7	10
C	C120	93.33	76.67	86.67	93.33	115	104	1.33	2.17	1.58	1.88	11	11	8	10

Table 1 the individual data of all participants on all administrated tests.

[1] A1 = amusics with abnormally high pitch discrimination thresholds, A2 = amusics with normal pitch discrimination thresholds, C = control participants.

[2] Scores of melodic MBEA were shown with the unit of percent.

[3] IQ = scaled scores of Metrics subtest of KBIT-2.

[4] Reading = composite index of TOWRE-2.

[5] Pitch thresholds = pitch discrimination thresholds with the unit of log10 cents.

[6] Rhythm = d' scores on each rhythmic condition and composite scores.

[7] Phonology = scaled scores on subtests of CTOPP-2. Digits = Memory for Digits subtest, Non-word = Non-word repetition subtest, RAN = Rapid Digit Naming subtest.

* marked the six amuscis who performed extremely poor on the simple condition of the rhythm task.
